# Utility of BerEp4/calretinin and desmin/epithelial membrane antigen (EMA) dual immunocytochemical staining in effusion cytology

**DOI:** 10.1002/cam4.5353

**Published:** 2022-10-19

**Authors:** Anders Hjerpe, Enes Demir, Sulaf Abd‐Own, Katalin Dobra

**Affiliations:** ^1^ Division of Pathology, Department of Laboratory Medicine Karolinska Institutet Stockholm Sweden; ^2^ Faculty of Medicine Eskisehir Osmangazi University Eskisehir Turkey

**Keywords:** cytology, diagnosis, dual stain, immunocytochemistry, mesothelioma

## Abstract

**Background:**

Pleural mesothelioma (PM) is typically diagnosed late during the disease. Earlier detection can increase the chance of effective therapy. Recurrent pleural effusions are the earliest symptoms displaying an array of cytomorphological changes from reactive atypia to malignancy. Diagnosis is possible on effusion cytology by applying molecular and immunocytochemical markers, the main difficulty being when to suspect PM and to differentiate PM from metastatic adenocarcinoma and reactive mesothelial proliferations.

**Methods:**

We evaluated the diagnostic performance of two immunocytochemical dual stains (BerEp4/Calretinin and Desmin/Epithelial Membrane Antigen (EMA)) on 149 ethanol‐fixed cytospin preparation as an initial step to solve the mentioned diagnostic difficulty. The immunocytochemical reactivity pattern was evaluated by two independent investigators. The final diagnosis corresponded to PM (*n* = 20), metastatic adenocarcinoma (*n* = 83), and mesotheliosis (*n* = 46) in these cases.

**Results:**

Calretinin had 99% specificity and 98% sensitivity for indicating a mesothelial phenotype, while BerEp4 distinguished the adenocarcinoma cases with 98% specificity and 99% sensitivity. EMA displayed 96% specificity and 99% sensitivity in malignant cases, while Desmin without EMA present showed 99% specificity and 96% sensitivity for indicating benign mesothelial proliferation.

**Conclusions:**

Interpretation of the four immunoreactions is improved when performed as dual stains. The dual staining is a useful tool in the initial handling of atypical effusions and guides the subsequent choice of antibody panels for more detailed subclassification of malignant effusions.

## Graphical abstract

We evaluated the diagnostic performance of two immunocytochemical dual stains BerEp4/Calretinin and Desmin/Epithelial Membrane Antigen (EMA) on 149 ethanol‐fixed cytospin preparation as an initial step to differentiate pleural mesothelioma from metastatic adenocarcinoma and reactive mesothelial proliferations. Calretinin had 99% specificity and 98% sensitivity for indicating mesothelial phenotype. BerEp4 distinguished the adenocarcinoma cases with 98% specificity and 99% sensitivity. EMA displayed 96% specificity and 99% sensitivity in malignant cases. Desmin showed 99% specificity and 96% sensitivity for indicating benign mesothelial proliferation. The results clearly indicate improved diagnostic performance of the four combined antibodies with the present dual staining design compared to previously published results based on single immunostainings. Our data prove the hypothesis that stronger typical reactivities override weaker nonspecific reactions facilitating identification of a true alien‐ and malignant cell population side by side with the tissue‐resident normal mesothelial cells.

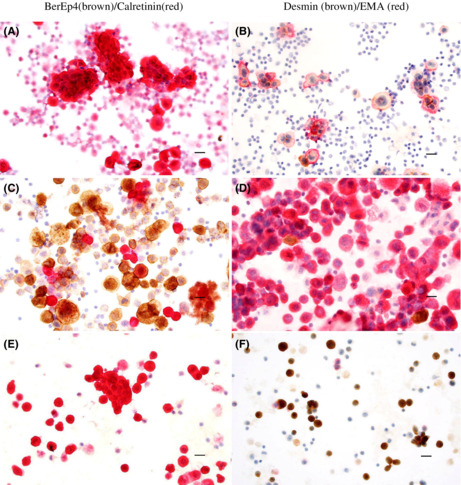

## 1 INTRODUCTION

Mesothelioma occurs in the thin mesothelial layer that surrounds the visceral organs. The most common form is pleural mesothelioma (PM) affecting the pleura due to asbestos exposure. Although there are various treatment options, they are most often not applicable because of the advanced stage of the disease at diagnosis. Considering this fact, early and noninvasive diagnosis of PM by effusion cytology can affect the prognosis positively.

The first sign of PM is a recurrent hemorrhagic effusion. The diagnosis can be reliably obtained by cytological evaluation of the effusion using adjuvant analyses such as molecular markers,^1^ immunocytochemistry, ploidy analysis (fluorescence in situ hybridization),^2^, ^3^ and electron microscopy, for detailed guidelines see.^4^ It has been shown in two independent audits^5^, ^6^ that the diagnosis can be established by cytology with sufficient sensitivity also when used in long‐term clinical routine. Since the effusion is the first available biologic material, the diagnosis can be obtained earlier by cytology than by conventional biopsy sampling.^7^ Studies also show that therapy is more efficient in the earlier stages of PM,^8^ thus treatment should be initiated without delay.

Effusions showing cellular atypia, high cellularity, and presence of papillary structures should raise the suspicion of a malignant condition. The main differential diagnoses consist of PM, metastatic adenocarcinoma, or reactive mesothelial proliferation (mesotheliosis [RM]). The cellular atypia in malignant conditions often shows considerable overlap with RM, and the use of immunocytochemistry will be helpful in effusions with abnormalities of uncertain significance.

As an initial step, the liberal use of a limited number of immunocytochemical stains improves the sensitivity of diagnosing PM. Immunocytochemistry will in these cases not only establish malignancy but also indicate the origin of a metastatic tumor and provide information for the choice of therapy. Depending on whether it is an PM or metastatic condition, different diagnostic antibody panels are used. To facilitate the initial step of deciding the cell lineage and choice of further diagnostic panels we optimized and applied two dual stains BerEp4/Calretinin and Desmin/EMA in this study.

Calretinin shows the mesothelial origin, whereas BerEp4 demonstrates the presence of an alien malignant epithelial cell population.^9^ The second combination includes Desmin and EMA.^10^, ^11^ Desmin reactivity is seen in benign mesothelial cells, and it disappears in malignant conditions, meanwhile EMA will indicate malignancy. Recently the alternate use of BAP1 instead of Desmin has been encouraged. However, the Desmin epitope seems to be sensitive to aldehyde fixations, and it seems to perform better than BAP1 when applied on ethanol‐fixed materials.

The aim of this study is to evaluate the diagnostic performance of these antibody combinations in a prospective manner.

## 2 MATERIALS AND METHODS

We have previously presented an audit of diagnosing PM by effusion cytology,^5^ most of the immunocytochemistry in that study being performed as single stains. All effusions in the present study were retrieved from the archives at the Laboratory for Clinical Cytology and Pathology of Karolinska University Hospital Huddinge. The two dual stains had been performed in clinical routine from January 1, 2019 to June 30, 2020, where a final diagnosis was either carcinoma, PM, or RM. The latter diagnosis group was identified as cases with no sign of cancer within 1 year after thoracocentesis. The altogether 149 samples thus examined were from 83 adenocarcinomas and 20 mesotheliomas while 46 cases were classified as RM with no subsequent diagnosis of malignancy. Three cases lacking definite diagnosis (malignancy present elsewhere but involvement of the pleura uncertain) were excluded. The study was approved by the ethical review board of Stockholm, Sweden (2007/1089–32).

**FIGURE 1:**
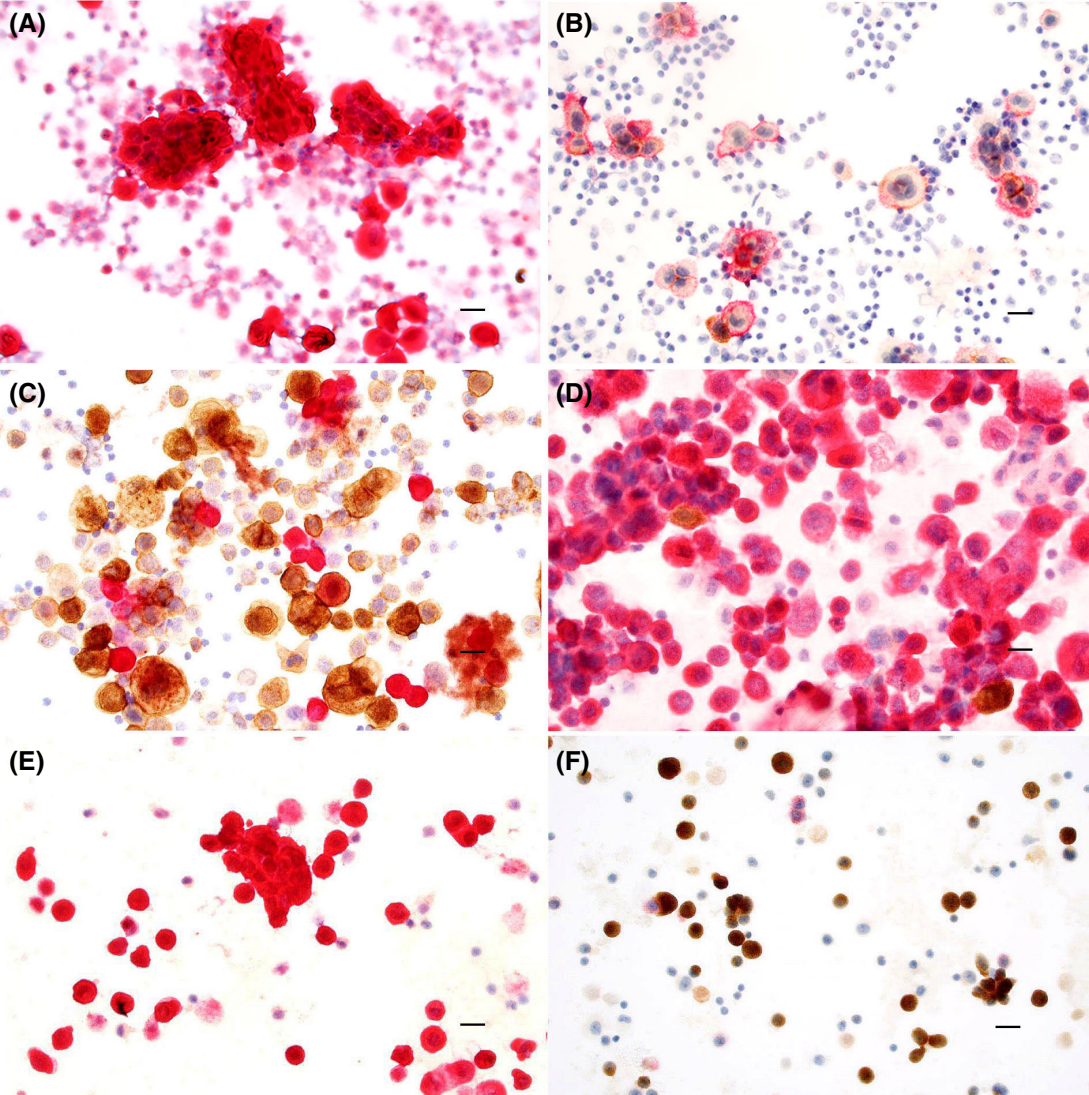
Reaction patterns obtained with dual staining of ethanol‐fixed effusions, combining in the left column (A, C, E) BerEp4 (brown) with Calretinin (red) and in the right column (B, D, F) Desmin (brown) with EMA (red). Mesothelioma is indicated by reactivity to Calretinin (A, red) and EMA (B, red), the latter stain often accentuated close to the cell membrane, while adenocarcinoma reacts to BerEp4 (C, brown) and EMA (D, red). Benign mesothelium binds the Calretinin antibody (E, red) and has retained its Desmin (F, brown). Bar = 20 μm. EMA, epithelial membrane antigen.

All immunostainings were performed as dual stains on ethanol‐fixed cytospin preparations, using the cytology dual staining program in the Leica Bond immunostainer (BOND‐III). Antibodies (DAKO, Copenhagen Denmark) were diluted as shown in Table 1. In this way, BerEp4 was combined with Calretinin and Desmin with EMA. Stainings employed the second step with peroxidase (BerEp4 and Desmin) or alkaline phosphatase (Calretinin and EMA) as labeling enzymes. Reaction products were visualized using Leica's chromophore reagents, yielding brown color for BerEp4 and Desmin and red for Calretinin and EMA. With the present antibodies Calretinin and Desmin reactivities were cytoplasmic as was that of EMA in carcinomas, while BerEp4 and EMA in PM also showed accentuation toward the cell membrane. Only distinct, moderate to strong BerEp4 imstaining of the alien epithelial cell population was considered as positive Figure 1, whereas weak BerEp4 reactivity present in macrophages was regarded as nonspecific.

**TABLE 1:** Antibodies and the features of antibodies used in dual staining on ethanol‐fixed cytospin preparations

Antibodies	Source	Original protein concentration	Dilution
Calretinin	Dako, M7245	266 mg/L	1:1000
BerEp4	Dako, M0804	100 mg/L	1:50
Desmin	Dako, M3575	86 mg/L	1:800
EMA	Dako, M0613	3700 mg/L	1:40

Abbreviation: EMA, epithelial membrane antigen.

All stainings had been performed in clinical routine. After retrieving the slides from the laboratory archive, they were all blindly evaluated by two observers. In case of discrepant opinions or any kind of uncertainty, the sample was discussed with a third person. In this evaluation, the samples were classified according to (1) presence of Calretinin without BerEp4 reactivity, (2) presence of reactivity to BerEp4, (3) presence of EMA reactivity, and (4) presence of Desmin staining without simultaneous EMA reactivity. Only distinct reactivities were considered after background subtraction. In the diagnostic process, the following evaluation algorithm has been applied: the combination of Calretinin and Desmin identifies reactive mesothelial cells. BerEp4 positivity corresponds to an “alien” true epithelial cell population normally not present in the serosal cavity and its presence indicates metastatic adenocarcinoma cells. EMA indicates PM in combination with Calretinin and adenocarcinoma in combination with BerEp4. The diagnostic utility of these four staining patterns was evaluated and their specificity, sensitivity, positive predictive value, and negative predictive value were calculated.

## 3 RESULTS

The presence of reactivities to the four different antibodies are shown in Table 2, and the calculated diagnostic performances in Table 3. The results indicate that the two dual stains provide useful information for the further handling of effusion cytology. Reactivity to Calretinin is supposed to show cells of mesothelial lineage and distinct reactivity without simultaneous EMA was found in 98% of samples from patients with PM or RM. The presence of cells with distinct reactivity to BerEp4, as an indication of an alien malignant cell population, was seen in 99% of effusions due to metastatic involvement of the pleura but only in one of the reactive conditions and in none of the PMs, giving a positive predictive value of 99%. EMA reactive cells in the effusion were present in 99% of the malignant effusions with a positive predictive value of 98%, that is, EMA reactivity was registered in 2 cases without any connection to malignancy. The immunostaining of Desmin without simultaneous EMA was also highly informative when applied to cells not fixed in formalin. Distinct Desmin reactivity was detected in 96% of the samples with mesotheliosis and in only one of the 103 malignant cases, giving a PPV of 98%.

**TABLE 2:** The proportion of reactivities of immunocytochemical stains in ethanol‐fixed cytospin effusions which consists of 20 pleural mesotheliomas (PMs), 83 carcinoma, and 46 mesotheliosis cases

Reactivity patterns	Pleural mesothelioma (PM)	Carcinoma	Mesotheliosis
Calretinin reactivity without BerEp4 reactivity	20/20 (100%)	1/83 (1%)	45/46 (98%)
BerEp4 reactivity	0/20 (0%)	82/83 (99%)	1/46 (2%)
EMA reactivity	19/20 (95%)	83/83 (100%)	2/46 (4%)
Desmin reactivity without simultaneous EMA reactivity	1/20 (5%)	0/83 (0%)	44/46 (96%)

Abbreviations: EMA, epithelial membrane antigen; PM, pleural mesothelioma.

**TABLE 3:** Sensitivity, specificity, positive predictive value, and negative predictive value for the four antibodies used

Reactivity patterns	Sensitivity	Specificity	PPV	NPV
Positive Calretinin indicating mesothelial phenotype	65/66 (98%)	82/83 (99%)	65/66 (98%)	82/83 (99%)
Positive BerEp4 indicating carcinoma	82/83 (99%)	65/66 (98%)	82/83 (99%)	65/66 (98%)
Positive EMA indicating malignancy	102/103 (99%)	44/46 (96%)	102/104 (98%)	44/45 (98%)
Positive Desmin without simultaneous EMA reactivity, indicating benign mesothelium	44/46 (96%)	102/103 (99%)	44/45 (98%)	102/104 (98%)

Abbreviations: EMA, epithelial membrane antigen; NPV, negative predictive value; PPV, positive predictive value.

## 4 DISCUSSION

The results clearly indicate improved diagnostic performance of the four combined antibodies with the present dual staining design compared to previously published results based on single immunostainings.^12^, ^13^, ^14^, ^15^, ^16^, ^17^ Our data prove the hypothesis that stronger typical reactivities override weaker nonspecific background cross reaction facilitating identification of a true alien‐ and malignant cell population side by side with the tissue‐resident normal mesothelial cells.

Of importance here for the use of Desmin, is that the immunocytochemistry is performed on cytospin preparations not exposed to formalin fixation, which gives a performance of Desmin better than that of the often‐suggested alternative BAP1. In a prior study^18^ the sensitivity of BAP1, in differentiating benign versus malignant mesothelial proliferation in cytospin (supplied from effusions) and cell block preparations, has been calculated as 86% and 88%, respectively. Another study,^19^ which comprises 147 pleural and peritoneal effusions including mesothelioma, adenocarcinoma, and benign mesothelial hyperplasia, investigated the loss of BAP1 expression for the diagnosis of mesothelioma by immunohistochemistry showed that loss of BAP1 occurs in 76.5% of the cell block samples and 47.5% of the biopsies in mesothelioma. Desmin has in this study performed well with the sensitivity of 96%, specificity of 99%, and 98% positive predictive value for indicating benign mesothelium with the help of EMA combination in dual staining. This result clearly shows that we have managed to obtain a high sensitivity with Desmin‐EMA dual stain in differentiating benign and malignant proliferations. In the present material, Desmin was more effective than previously had been demonstrated with BAP1. It may be that BAP1 can replace Desmin when using paraffin‐embedded cell blocks, but this remains to be shown.

Calretinin labels mesothelial cells while BerEp4 is generally used for indicating carcinomas as single stains. However, in single staining, BerEp4 reactivity can occasionally be expressed also in mesothelial cells. Thus, BerEp4 showed reactivity in two samples in a prior effusion immunocytochemistry study,^20^ which includes 15 mesothelial proliferation (14 benign and one malignant) in cytospin preparations. Another study^21^ indicated that BerEp4 was weakly reactive in three RM cases and two mesothelioma cases. In this latter study, the sensitivity and specificity of BerEp4 for indicating adenocarcinoma were only 76.4% and 86.8%, while the corresponding measures in the present study were 99% and 98%, respectively. The use of Ber Ep4 as a single stain will, thus, have less diagnostic accuracy and these kinds of weak expressions can cause interpretation errors or confusion.

One of the advantages of BerEp4/Calretinin dual staining becomes apparent here. When combining the two markers in the dual staining, Calretinin is almost invariably stronger in mesothelial cells, hiding a possible weaker BerEp4 reaction. Furthermore, the sensitivity and specificity for Calretinin, indicating a mesothelial phenotype were 98% and 99% with the present dual staining technique. The distinction of adenocarcinoma from PM in effusions could in a previous meta‐analysis^22^ be performed with 84% sensitivity and 96% specificity, using Calretinin and BerEp4 as single stains. Separation of the two different populations ‐ the mesothelial versus adenocarcinoma populations – side by side on the same slide is, therefore, more accurate, using the dual staining design.

Similar to the possible reactivity of BerEp4 in the mesothelial cells, immunoreactivity of EMA can also sometimes be observed in the mesothelial cells. A study^23^ that investigates the diagnostic usefulness of various markers including EMA and includes 171 effusion cytospin preparations (87 pleural effusion cases and 84 peritoneal cases including 50 RM, 11mesotheliomas, and 110 metastatic adenocarcinomas) showed that EMA was reactive in 13/50 RM samples (26%). The results in that study^23^ indicate that the positive predictive value of single stain EMA for indicating malignancy was 120/133 (90%), while sensitivity and specificity were 99% and 74%, respectively. With the dual staining, possible aberrant expressions like immunoreactivity of EMA in RM cases can be hidden by stronger Desmin reactivity, overriding the weaker reaction in favor of dominancy, explaining why EMA reactivity indicated malignancy with 98% PPV, 99% sensitivity, and 96% specificity.

The two dual stains will in most malignant cases not be sufficient for a final diagnosis, but as an initial step, they provide important information regarding the choice of subsequent diagnostic analyses. A specimen only containing cells positive to Calretinin and Desmin can motivate a final diagnosis of a reactive condition without association to malignancy. With cells showing distinct reactivity to BerEp4, this indicates the presence of carcinoma, and the subsequent diagnostic effort should be directed toward establishing the primary location of the tumor, and when appropriate genetic analyses are relevant to the choice of treatment.

When, on the other hand, the cells co‐express EMA and Calretinin, then this may indicate PM. The International Guidelines^4^ states that four immunocytochemical reactions could be sufficient for the diagnosis of PM. However, in most cases, it is wise to use some additional reactions. Thus, this reaction pattern motivates subsequent immunocytochemistry with a panel that may establish the PM diagnosis, including other mesothelial markers such as WT1, CK5, D2‐40, HBME‐1 when working with cytospin preparations or combining BAP‐1 and MTAP after cell block preparation. Similarly, additional marker excluding PM such monoclonal CEA, MOC31, Claudin 4 and TTF1 can be useful. One of the present effusions contained unequivocally malignant cells that were reactive to both Calretinin and EMA while BerEp4 and Desmin were negative. However, the further follow‐up lacked all other mesothelial markers, excluding the alternative of PM, and the final diagnosis was carcinosarcoma.

In questionable cases with less distinct reaction patterns, other adjuvant analyses can be helpful. Thus, ploidy analysis by fluorescence in situ hybridization may help in establishing a malignant nature of the cells. Other adjuvant measures aiming at diagnosing PM can be the chemical analysis of biomarkers such as hyaluronan and mesothelin^24^ and, as the final attempt electron microscopy of a cell pellet.^25^, ^26^, ^27^

These dual stains can be also used in other effusions. Although, the frequent need of other adjuvant analyses is recommended before the definite diagnosis.^4^

## 5 CONCLUSION

The four presented immunocytochemical markers are useful in effusion cytology when used as dual stains for the diagnosis of pleural mesothelioma and distinguishing it from carcinoma and reactive mesotheliosis. The use of the dual staining technique will decrease the risk of overinterpretation of weaker reactivities of BerEp4 and EMA that can be seen in reactive mesothelial cells. In clinical routine, the reactions are preferably performed as an initial step to guide the choice of further diagnostic measures. With liberal use of these stainings, cases with reactive mesothelial proliferation are readily identified, and when indicating malignancy, the obtained pattern will indicate a suitable subsequent choice of immunopanels.

## AUTHOR CONTRIBUTIONS

**Anders Hjerpe:** Conceptualization (equal); formal analysis (lead); investigation (lead); methodology (equal); supervision (equal); validation (equal); visualization (equal); writing – original draft (equal). **Enes Demir:** Data curation (supporting); formal analysis (supporting); investigation (supporting); visualization (lead); writing – review and editing (supporting). **Sulaf Abd‐Own:** Methodology (supporting); writing – review and editing (supporting). **Katalin Dobra:** Conceptualization (equal); funding acquisition (lead); investigation (supporting); methodology (supporting); project administration (lead); resources (equal); supervision (lead); writing – review and editing (lead).

## FUNDING INFORMATION

The study has been supported by the Cancer Society in Stockholm and the King Gustaf V Jubilee Fund.

## CONFLICT OF INTEREST

Authors made no disclosures.

## Data Availability

Data sharing is not applicable to this article as no new data were created or analyzed in this study.
